# Crystal structure of ethyl 5′′-fluoro-2′′,3-dioxo-6′,7′,8′,8a’-tetra­hydro-2′*H*,3*H*,5′*H*-di­spiro­[benzo[*b*]thio­phene-2,1′-indol­izine-3′,3′′-indoline]-2′-carboxyl­ate

**DOI:** 10.1107/S2056989015002121

**Published:** 2015-02-07

**Authors:** R. Raja, J. Govindaraj, M. Suresh, R. Raghunathan, A. SubbiahPandi

**Affiliations:** a Department of Physics, Presidency College (Autonomous), Chennai 600 005, India; b Department of Physics, Pachaiyappa’s College for Men, Kanchipuram 631 501, India; c Department of Organic Chemistry, University of Madras, Guindy Campus, Chennai 602 025, India

**Keywords:** crystal structure, di­spiro, benzo­thio­phene, indolizine, indoline, F⋯F inter­actions, hydrogen bonds

## Abstract

In the title compound, C_25_H_23_FN_2_O_4_S, the fused piperidine ring of the octa­hydro­indolizine ring system adopts a chair conformation and the five-membered ring has a twisted conformation on the N—C(spiro) bond. The mean planes of the benzo­thio­phene and indoline ring systems are inclined to the mean plane of the pyrrolidine ring by 83.1 (1) and 84.9 (1)°, respectively, and to each other by 29.37 (17)°. In the crystal, mol­ecules are linked *via* pairs of N—H⋯O hydrogen bonds, forming inversion dimers with an *R*_2_^2^(8) ring motif. The dimers are linked *via* C—H⋯O hydrogen bonds, forming slabs lying parallel to (100). The packing between the slabs features a short [2.734 (2) Å] F⋯F contact.

## Related literature   

For the biological activity of indole derivatives, see: Barden (2011 ▸); Oudard *et al.* (2011 ▸); Beale (2011 ▸); Aanandhi *et al.* (2008 ▸); Muthukumar *et al.* (2008 ▸). For crystal structures of similar compounds, see: Savithri *et al.* (2014 ▸).
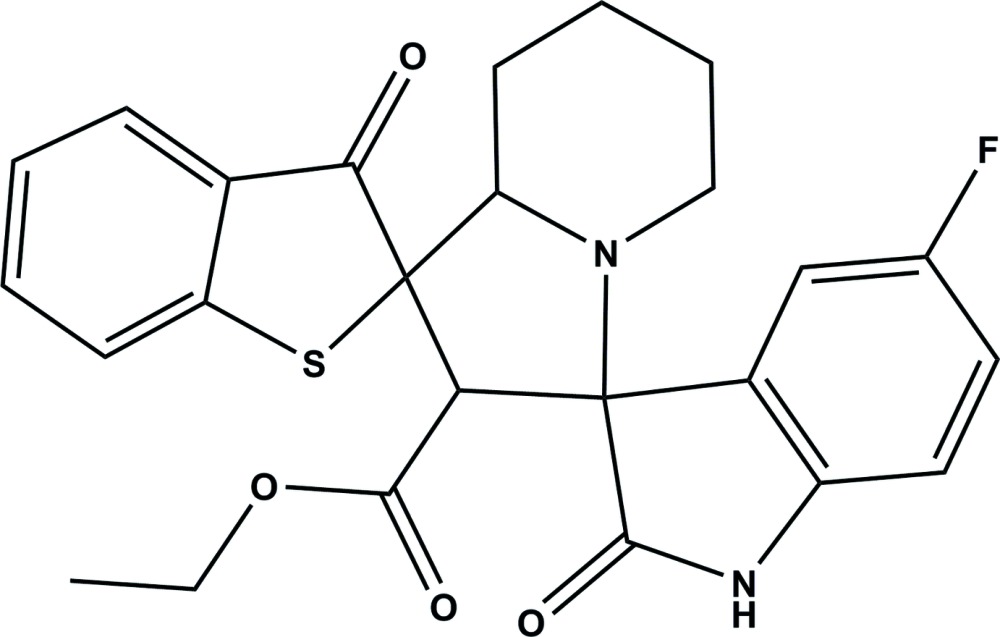


## Experimental   

### Crystal data  

C_25_H_23_FN_2_O_4_S
*M* *_r_* = 466.51
Monoclinic, 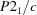

*a* = 13.877 (2) Å
*b* = 11.8999 (19) Å
*c* = 15.426 (4) Å
β = 116.463 (4)°
*V* = 2280.5 (8) Å^3^
*Z* = 4
Mo *K*α radiation
μ = 0.19 mm^−1^
*T* = 293 K
0.30 × 0.30 × 0.30 mm

### Data collection  

Bruker SMART APEXII area-detector diffractometer
Absorption correction: multi-scan (*SADABS*; Bruker, 2008 ▸) *T*_min_ = 0.947, *T*_max_ = 0.955
32121 measured reflections
4784 independent reflections
3652 reflections with *I* > 2σ(*I*)
*R*_int_ = 0.033

### Refinement  

*R*[*F* ^2^ > 2σ(*F* ^2^)] = 0.044
*wR*(*F* ^2^) = 0.119
*S* = 1.04
4784 reflections
303 parameters
H atoms treated by a mixture of independent and constrained refinement
Δρ_max_ = 0.38 e Å^−3^
Δρ_min_ = −0.24 e Å^−3^

### 

Data collection: *APEX2* (Bruker, 2008 ▸); cell refinement: *SAINT* (Bruker, 2008 ▸); data reduction: *SAINT*; program(s) used to solve structure: *SHELXS97* (Sheldrick, 2008 ▸); program(s) used to refine structure: *SHELXL2014* (Sheldrick, 2015 ▸); molecular graphics: *PLATON* (Spek, 2009 ▸); software used to prepare material for publication: *SHELXL2014* and *PLATON* (Spek, 2009 ▸).

## Supplementary Material

Crystal structure: contains datablock(s) global, I. DOI: 10.1107/S2056989015002121/su5063sup1.cif

Structure factors: contains datablock(s) I. DOI: 10.1107/S2056989015002121/su5063Isup2.hkl

**Fig. 1.**
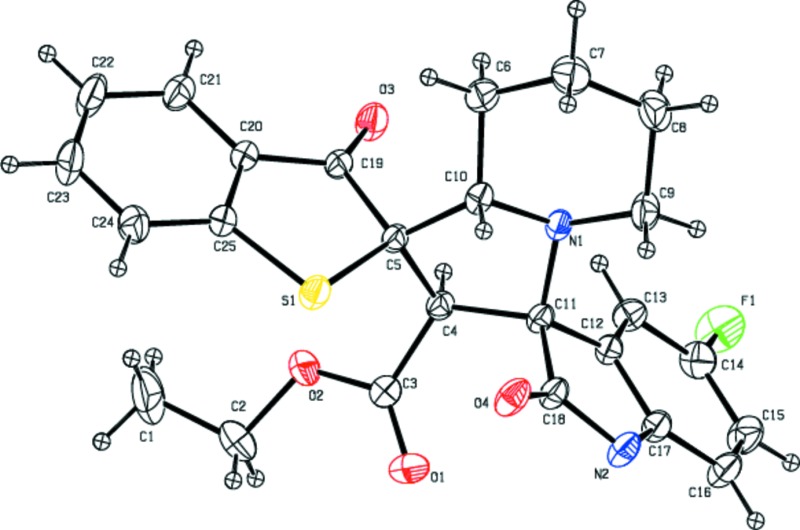
The molecular structure of the title compound, showing the atom labelling. Displacement ellipsoids are drawn at the 30% probability level.

**Fig. 2.**
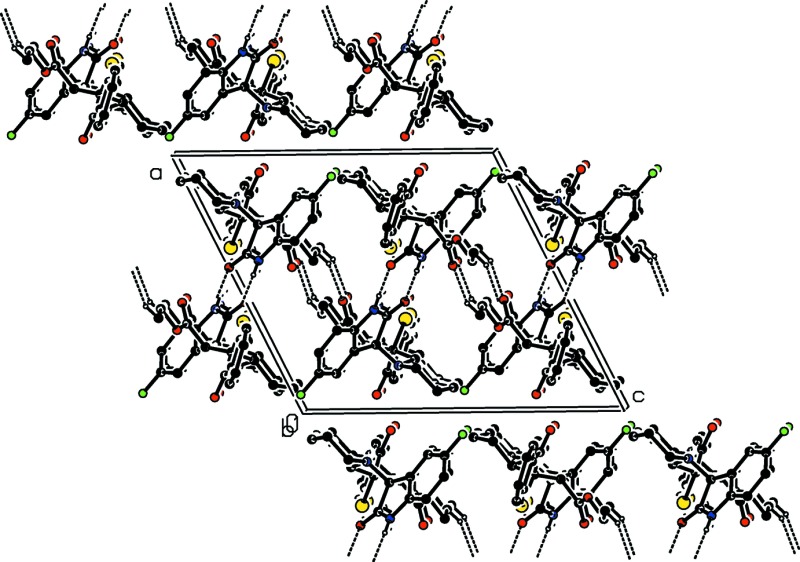
A view along the b axis of the crystal packing of the title compound. Hydrogen bonds are shown as dashed lines (see Table 1 for details; H atoms not involved in these interactions have been omitted for clarity).

. DOI: 10.1107/S2056989015002121/su5063fig1.tif
The mol­ecular structure of the title compound, showing the atom labelling. Displacement ellipsoids are drawn at the 30% probability level.

b . DOI: 10.1107/S2056989015002121/su5063fig2.tif
A view along the *b* axis of the crystal packing of the title compound. Hydrogen bonds are shown as dashed lines (see Table 1 for details; H atoms not involved in these inter­actions have been omitted for clarity).

CCDC reference: 1046671

Additional supporting information:  crystallographic information; 3D view; checkCIF report

## Figures and Tables

**Table 1:** Hydrogen-bond geometry (, )

*D*H*A*	*D*H	H*A*	*D* *A*	*D*H*A*
N2H2O4^i^	0.87(3)	1.96(3)	2.834(2)	179(2)
C1H1*A*O1^ii^	0.96	2.50	3.401(4)	156

Symmetry codes: (i) 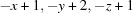; (ii) 
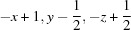
.

## supplementary crystallographic information

### S1. Comment

Indole-containing compounds are best known for their medicinal properties in the pharmaceutical industry. In modern times, analogs based on indole are significant players in a diverse array of markets such as dyes, plastics, agriculture, vitamin supplements, over-the-counterdrugs, flavour enhancers and perfumery (Barden, 2011). Several indole derivatives, such as Sunitinib, a tyrosine kinase inhibitor (Oudard *et al.*, 2011), or Delavirdine a non-nucleoside reverse transcriptase inhibitor (Beale, 2011), are in clinical use. Spiroindoles are important heterocyclic compounds with diverse bioactivities (Aanandhi *et al.*, 2008; Muthukumar *et al.*, 2008).

The X-ray study confirmed the molecular structure and atomic connectivity for the title compound, as illustrated in Fig. 1. Pyridine ring adopts a chair conformation [puckering parameters q_2_= 0.069 (3) Å and π_2_= 303 (2)°]. The pyrrole ring adopts a twisted conformation with the lowest asymmetry parameters ΔC2(N1—C10) = 1.8 (2)°. The pyrrole ring system is oriented with a dihedral angles of 84.9 (1) and 83.1 (1)°, respectively with respect to the mean planes of benzothiophene ring and indole ring systems.

In the crystal, molecules are linked via N-H···O hydrogen bonds forming inversion dimers with an R^2^_2_(8) ring motif (Table 1 and Fig. 2). The dimers are linked via C—H···O hydrogen bonds forming slabs lying parallel to (100); see Table 1 and Fig. 2. The slabs are linked by a short F···F^i^ interaction [2.73482) Å, symmetry code: (i) -x, -y+2, -z] forming a three-dimensional structure.

### S2. Experimental

A reaction mixture of (*E*)-ethyl 2-(3-oxobenzo[*b*]thiophen-2(3*H*)-ylidene)acetate (1.0 mmol), 5-Fluoroisatin (1.1 mmol) and pipecolic acid (1.1 mmol) was refluxed in methanol (20 ml) until completion of the reaction was evidenced by TLC analysis. After completion of the reaction the solvent was evaporated under reduced pressure. The crude reaction mixture was dissolved in dichloromethane (2 *x* 50 ml) and washed with water followed by brine solution. The organic layer was separated and dried over sodium sulfate. After filtration and evaporation of the organic solvent was carried out under reduced pressure. The product was separated by column chromatography using hexane and ethyl acetate (9:1) as an eluent to give a colorless solid. The product was dissolved in chloroform (3 ml) and heated for two minutes. The resulting solution was subjected to crystallization by slow evaporation of the solvent resulting in single crystals suitable for X-ray crystallographic studies.

### S3. Refinement

The NH H atom was located in a difference Fourier map and freely refined. The C-bound H atoms were positioned geometrically (C–H = 0.93–0.98 Å) and allowed to ride on their parent atoms, with *U*_iso_(H) = 1.5*U*_eq_(C) for methyl H atoms and = 1.2*U*_eq_(C) for all other H atoms.

### Crystal data

**Table:**

C_25_H_23_FN_2_O_4_S	*F*(000) = 976
*M**_r_* = 466.51	*D*_x_ = 1.359 Mg m^−^^3^
Monoclinic, *P*2_1_/*c*	Mo *K*α radiation, λ = 0.71073 Å
*a* = 13.877 (2) Å	Cell parameters from 3238 reflections
*b* = 11.8999 (19) Å	θ = 1.6–25.0°
*c* = 15.426 (4) Å	µ = 0.19 mm^−^^1^
β = 116.463 (4)°	*T* = 293 K
*V* = 2280.5 (8) Å^3^	Block, colourless
*Z* = 4	0.30 × 0.30 × 0.30 mm

### Data collection

**Table:**

Bruker SMART APEXII area-detector diffractometer	4784 independent reflections
Radiation source: fine-focus sealed tube	3652 reflections with *I* > 2σ(*I*)
Graphite monochromator	*R*_int_ = 0.033
ω and φ scans	θ_max_ = 26.6°, θ_min_ = 1.6°
Absorption correction: multi-scan (*SADABS*; Bruker, 2008)	*h* = −17→17
*T*_min_ = 0.947, *T*_max_ = 0.955	*k* = −15→15
32121 measured reflections	*l* = −19→19

### Refinement

**Table:**

Refinement on *F*^2^	0 restraints
Least-squares matrix: full	Hydrogen site location: mixed
*R*[*F*^2^ > 2σ(*F*^2^)] = 0.044	H atoms treated by a mixture of independent and constrained refinement
*wR*(*F*^2^) = 0.119	*w* = 1/[σ^2^(*F*_o_^2^) + (0.0484*P*)^2^ + 1.3589*P*] where *P* = (*F*_o_^2^ + 2*F*_c_^2^)/3
*S* = 1.03	(Δ/σ)_max_ = 0.001
4784 reflections	Δρ_max_ = 0.38 e Å^−^^3^
303 parameters	Δρ_min_ = −0.24 e Å^−^^3^

### Special details

**Table:**

Geometry. All esds (except the esd in the dihedral angle between two l.s. planes) are estimated using the full covariance matrix. The cell esds are taken into account individually in the estimation of esds in distances, angles and torsion angles; correlations between esds in cell parameters are only used when they are defined by crystal symmetry. An approximate (isotropic) treatment of cell esds is used for estimating esds involving l.s. planes.

### Fractional atomic coordinates and isotropic or equivalent isotropic displacement parameters (Å^2^)

**Table:**

	*x*	*y*	*z*	*U*_iso_*/*U*_eq_	
S1	0.35506 (4)	0.59504 (4)	0.45922 (4)	0.04126 (16)	
F1	0.07518 (12)	1.08370 (13)	0.02567 (10)	0.0707 (5)	
O1	0.42901 (14)	0.75803 (15)	0.29676 (15)	0.0677 (5)	
O2	0.32609 (12)	0.60522 (12)	0.24108 (12)	0.0484 (4)	
O3	0.06507 (11)	0.61184 (12)	0.25149 (11)	0.0497 (4)	
O4	0.42650 (12)	0.85900 (12)	0.48990 (10)	0.0464 (4)	
N1	0.18102 (13)	0.86296 (13)	0.36986 (11)	0.0320 (4)	
N2	0.39881 (14)	1.00547 (14)	0.38496 (13)	0.0413 (4)	
H2	0.453 (2)	1.047 (2)	0.4226 (18)	0.055 (7)*	
C1	0.3758 (4)	0.4290 (3)	0.2058 (4)	0.1166 (15)	
H1A	0.4227	0.3898	0.1854	0.175*	
H1B	0.3032	0.4248	0.1557	0.175*	
H1C	0.3799	0.3950	0.2638	0.175*	
C2	0.4091 (2)	0.5476 (2)	0.2250 (2)	0.0677 (8)	
H2A	0.4781	0.5538	0.2817	0.081*	
H2B	0.4155	0.5801	0.1701	0.081*	
C3	0.34861 (16)	0.70724 (17)	0.27985 (16)	0.0401 (5)	
C4	0.25734 (14)	0.74688 (15)	0.29925 (13)	0.0308 (4)	
H4	0.1921	0.7407	0.2377	0.037*	
C5	0.23753 (14)	0.67371 (15)	0.37302 (13)	0.0305 (4)	
C6	0.10876 (18)	0.73457 (18)	0.44935 (17)	0.0459 (5)	
H6A	0.1225	0.6662	0.4873	0.055*	
H6B	0.0443	0.7236	0.3890	0.055*	
C7	0.0934 (2)	0.8330 (2)	0.50504 (19)	0.0569 (6)	
H7A	0.0297	0.8203	0.5146	0.068*	
H7B	0.1548	0.8380	0.5683	0.068*	
C8	0.0813 (2)	0.9427 (2)	0.45076 (19)	0.0553 (6)	
H8A	0.0129	0.9426	0.3930	0.066*	
H8B	0.0806	1.0046	0.4914	0.066*	
C9	0.17105 (19)	0.96107 (17)	0.42185 (17)	0.0454 (5)	
H9A	0.1557	1.0270	0.3809	0.054*	
H9B	0.2383	0.9737	0.4792	0.054*	
C10	0.20346 (16)	0.76111 (15)	0.42910 (14)	0.0336 (4)	
H10	0.2660	0.7761	0.4912	0.040*	
C11	0.26183 (14)	0.86879 (15)	0.33304 (13)	0.0298 (4)	
C12	0.23884 (15)	0.95884 (15)	0.25758 (13)	0.0315 (4)	
C13	0.15370 (16)	0.97322 (17)	0.16708 (14)	0.0381 (5)	
H13	0.0970	0.9223	0.1421	0.046*	
C14	0.15659 (17)	1.06670 (19)	0.11540 (15)	0.0443 (5)	
C15	0.23724 (19)	1.14452 (19)	0.14926 (17)	0.0499 (6)	
H15	0.2349	1.2064	0.1116	0.060*	
C16	0.32277 (18)	1.13050 (18)	0.24034 (17)	0.0467 (5)	
H16	0.3788	1.1822	0.2652	0.056*	
C17	0.32165 (16)	1.03742 (16)	0.29245 (14)	0.0358 (4)	
C18	0.37352 (15)	0.90668 (16)	0.41243 (14)	0.0346 (4)	
C19	0.14945 (14)	0.58543 (15)	0.31808 (14)	0.0331 (4)	
C20	0.18285 (15)	0.47195 (16)	0.35464 (14)	0.0344 (4)	
C21	0.11991 (19)	0.37579 (17)	0.32308 (17)	0.0464 (5)	
H21	0.0485	0.3808	0.2767	0.056*	
C22	0.1637 (2)	0.27384 (19)	0.3607 (2)	0.0601 (7)	
H22	0.1221	0.2090	0.3404	0.072*	
C23	0.2707 (2)	0.26735 (18)	0.4297 (2)	0.0589 (7)	
H23	0.3002	0.1973	0.4537	0.071*	
C24	0.33419 (19)	0.36132 (18)	0.46330 (17)	0.0474 (5)	
H24	0.4053	0.3556	0.5101	0.057*	
C25	0.28901 (16)	0.46518 (16)	0.42540 (14)	0.0349 (4)	

### Atomic displacement parameters (Å^2^)

**Table:**

	*U*^11^	*U*^22^	*U*^33^	*U*^12^	*U*^13^	*U*^23^
S1	0.0304 (2)	0.0319 (3)	0.0474 (3)	−0.0023 (2)	0.0046 (2)	0.0043 (2)
F1	0.0645 (9)	0.0798 (11)	0.0452 (8)	−0.0036 (8)	0.0042 (7)	0.0238 (7)
O1	0.0545 (10)	0.0552 (10)	0.1114 (15)	−0.0150 (8)	0.0533 (11)	−0.0126 (10)
O2	0.0471 (9)	0.0390 (8)	0.0651 (10)	0.0031 (7)	0.0304 (8)	−0.0106 (7)
O3	0.0325 (7)	0.0351 (8)	0.0596 (10)	−0.0030 (6)	0.0009 (7)	−0.0007 (7)
O4	0.0420 (8)	0.0379 (8)	0.0404 (8)	−0.0110 (6)	0.0013 (6)	0.0069 (6)
N1	0.0370 (8)	0.0234 (8)	0.0375 (9)	−0.0013 (6)	0.0184 (7)	−0.0005 (6)
N2	0.0395 (9)	0.0313 (9)	0.0403 (9)	−0.0136 (8)	0.0063 (8)	0.0003 (7)
C1	0.159 (4)	0.0526 (19)	0.188 (4)	0.030 (2)	0.123 (4)	−0.005 (2)
C2	0.0676 (17)	0.0603 (16)	0.094 (2)	0.0148 (14)	0.0527 (16)	−0.0084 (15)
C3	0.0392 (11)	0.0346 (11)	0.0480 (12)	−0.0025 (9)	0.0208 (9)	−0.0010 (9)
C4	0.0295 (9)	0.0270 (9)	0.0330 (10)	−0.0038 (7)	0.0112 (8)	−0.0033 (7)
C5	0.0257 (9)	0.0241 (9)	0.0365 (10)	−0.0021 (7)	0.0093 (8)	0.0006 (7)
C6	0.0538 (13)	0.0395 (12)	0.0549 (13)	−0.0007 (10)	0.0336 (11)	0.0064 (10)
C7	0.0713 (17)	0.0557 (15)	0.0614 (15)	0.0034 (13)	0.0456 (14)	0.0020 (12)
C8	0.0738 (17)	0.0443 (13)	0.0642 (15)	0.0102 (12)	0.0455 (14)	−0.0025 (12)
C9	0.0647 (14)	0.0286 (10)	0.0512 (13)	0.0016 (10)	0.0333 (11)	−0.0036 (9)
C10	0.0379 (10)	0.0264 (9)	0.0347 (10)	−0.0021 (8)	0.0145 (8)	0.0017 (8)
C11	0.0301 (9)	0.0252 (9)	0.0304 (9)	−0.0041 (7)	0.0101 (8)	−0.0010 (7)
C12	0.0343 (10)	0.0264 (9)	0.0348 (10)	−0.0018 (8)	0.0162 (8)	−0.0013 (8)
C13	0.0374 (10)	0.0397 (11)	0.0349 (10)	−0.0035 (9)	0.0141 (9)	0.0007 (9)
C14	0.0432 (12)	0.0489 (13)	0.0354 (11)	0.0049 (10)	0.0127 (9)	0.0086 (10)
C15	0.0589 (14)	0.0420 (12)	0.0505 (13)	0.0003 (11)	0.0259 (11)	0.0169 (10)
C16	0.0495 (13)	0.0345 (11)	0.0534 (13)	−0.0098 (9)	0.0205 (11)	0.0053 (10)
C17	0.0377 (10)	0.0298 (10)	0.0389 (11)	−0.0031 (8)	0.0161 (9)	0.0013 (8)
C18	0.0351 (10)	0.0275 (9)	0.0364 (10)	−0.0064 (8)	0.0117 (8)	−0.0015 (8)
C19	0.0280 (9)	0.0267 (9)	0.0414 (11)	−0.0035 (7)	0.0126 (8)	−0.0040 (8)
C20	0.0347 (10)	0.0266 (9)	0.0427 (11)	−0.0019 (8)	0.0180 (9)	−0.0011 (8)
C21	0.0468 (12)	0.0335 (11)	0.0554 (13)	−0.0079 (9)	0.0198 (11)	−0.0049 (10)
C22	0.0697 (17)	0.0259 (11)	0.0760 (17)	−0.0098 (11)	0.0246 (14)	−0.0020 (11)
C23	0.0758 (17)	0.0253 (11)	0.0736 (17)	0.0053 (11)	0.0313 (14)	0.0067 (11)
C24	0.0468 (12)	0.0377 (11)	0.0536 (13)	0.0078 (10)	0.0186 (11)	0.0079 (10)
C25	0.0374 (10)	0.0273 (9)	0.0416 (11)	0.0004 (8)	0.0191 (9)	0.0015 (8)

### Geometric parameters (Å, º)

**Table:**

S1—C25	1.753 (2)	C7—H7A	0.9700
S1—C5	1.8362 (18)	C7—H7B	0.9700
F1—C14	1.356 (2)	C8—C9	1.515 (3)
O1—C3	1.191 (3)	C8—H8A	0.9700
O2—C3	1.328 (2)	C8—H8B	0.9700
O2—C2	1.454 (3)	C9—H9A	0.9700
O3—C19	1.205 (2)	C9—H9B	0.9700
O4—C18	1.228 (2)	C10—H10	0.9800
N1—C9	1.458 (2)	C11—C12	1.508 (3)
N1—C10	1.465 (2)	C11—C18	1.554 (2)
N1—C11	1.467 (2)	C12—C13	1.380 (3)
N2—C18	1.348 (2)	C12—C17	1.390 (3)
N2—C17	1.402 (3)	C13—C14	1.380 (3)
N2—H2	0.87 (3)	C13—H13	0.9300
C1—C2	1.474 (4)	C14—C15	1.365 (3)
C1—H1A	0.9600	C15—C16	1.387 (3)
C1—H1B	0.9600	C15—H15	0.9300
C1—H1C	0.9600	C16—C17	1.373 (3)
C2—H2A	0.9700	C16—H16	0.9300
C2—H2B	0.9700	C19—C20	1.457 (3)
C3—C4	1.501 (3)	C20—C21	1.389 (3)
C4—C11	1.533 (2)	C20—C25	1.391 (3)
C4—C5	1.552 (3)	C21—C22	1.365 (3)
C4—H4	0.9800	C21—H21	0.9300
C5—C19	1.547 (2)	C22—C23	1.391 (4)
C5—C10	1.555 (3)	C22—H22	0.9300
C6—C10	1.513 (3)	C23—C24	1.374 (3)
C6—C7	1.523 (3)	C23—H23	0.9300
C6—H6A	0.9700	C24—C25	1.391 (3)
C6—H6B	0.9700	C24—H24	0.9300
C7—C8	1.520 (3)		
			
C25—S1—C5	93.21 (9)	C8—C9—H9B	109.7
C3—O2—C2	117.57 (19)	H9A—C9—H9B	108.2
C9—N1—C10	111.42 (15)	N1—C10—C6	109.79 (16)
C9—N1—C11	116.84 (15)	N1—C10—C5	103.83 (15)
C10—N1—C11	107.09 (14)	C6—C10—C5	118.85 (16)
C18—N2—C17	111.63 (16)	N1—C10—H10	108.0
C18—N2—H2	123.8 (16)	C6—C10—H10	108.0
C17—N2—H2	124.2 (16)	C5—C10—H10	108.0
C2—C1—H1A	109.5	N1—C11—C12	113.40 (15)
C2—C1—H1B	109.5	N1—C11—C4	99.47 (14)
H1A—C1—H1B	109.5	C12—C11—C4	116.58 (15)
C2—C1—H1C	109.5	N1—C11—C18	112.01 (15)
H1A—C1—H1C	109.5	C12—C11—C18	101.29 (14)
H1B—C1—H1C	109.5	C4—C11—C18	114.69 (15)
O2—C2—C1	106.6 (2)	C13—C12—C17	119.70 (18)
O2—C2—H2A	110.4	C13—C12—C11	131.32 (17)
C1—C2—H2A	110.4	C17—C12—C11	108.98 (16)
O2—C2—H2B	110.4	C14—C13—C12	116.89 (19)
C1—C2—H2B	110.4	C14—C13—H13	121.6
H2A—C2—H2B	108.6	C12—C13—H13	121.6
O1—C3—O2	124.9 (2)	F1—C14—C15	117.2 (2)
O1—C3—C4	126.0 (2)	F1—C14—C13	118.98 (19)
O2—C3—C4	109.08 (17)	C15—C14—C13	123.8 (2)
C3—C4—C11	117.05 (16)	C14—C15—C16	119.4 (2)
C3—C4—C5	114.26 (16)	C14—C15—H15	120.3
C11—C4—C5	105.98 (15)	C16—C15—H15	120.3
C3—C4—H4	106.3	C17—C16—C15	117.5 (2)
C11—C4—H4	106.3	C17—C16—H16	121.2
C5—C4—H4	106.3	C15—C16—H16	121.2
C19—C5—C4	109.53 (15)	C16—C17—C12	122.62 (19)
C19—C5—C10	113.40 (15)	C16—C17—N2	127.73 (19)
C4—C5—C10	103.29 (14)	C12—C17—N2	109.64 (17)
C19—C5—S1	106.29 (12)	O4—C18—N2	125.60 (18)
C4—C5—S1	115.26 (13)	O4—C18—C11	125.97 (17)
C10—C5—S1	109.29 (13)	N2—C18—C11	108.26 (16)
C10—C6—C7	108.01 (18)	O3—C19—C20	126.31 (17)
C10—C6—H6A	110.1	O3—C19—C5	121.35 (17)
C7—C6—H6A	110.1	C20—C19—C5	112.32 (15)
C10—C6—H6B	110.1	C21—C20—C25	120.52 (18)
C7—C6—H6B	110.1	C21—C20—C19	125.85 (18)
H6A—C6—H6B	108.4	C25—C20—C19	113.60 (16)
C8—C7—C6	111.02 (19)	C22—C21—C20	119.5 (2)
C8—C7—H7A	109.4	C22—C21—H21	120.3
C6—C7—H7A	109.4	C20—C21—H21	120.3
C8—C7—H7B	109.4	C21—C22—C23	119.7 (2)
C6—C7—H7B	109.4	C21—C22—H22	120.1
H7A—C7—H7B	108.0	C23—C22—H22	120.1
C9—C8—C7	112.5 (2)	C24—C23—C22	122.0 (2)
C9—C8—H8A	109.1	C24—C23—H23	119.0
C7—C8—H8A	109.1	C22—C23—H23	119.0
C9—C8—H8B	109.1	C23—C24—C25	118.1 (2)
C7—C8—H8B	109.1	C23—C24—H24	120.9
H8A—C8—H8B	107.8	C25—C24—H24	120.9
N1—C9—C8	109.79 (18)	C20—C25—C24	120.17 (18)
N1—C9—H9A	109.7	C20—C25—S1	114.38 (14)
C8—C9—H9A	109.7	C24—C25—S1	125.45 (16)
N1—C9—H9B	109.7		
			
C3—O2—C2—C1	165.9 (3)	C4—C11—C12—C17	−128.26 (18)
C2—O2—C3—O1	5.0 (4)	C18—C11—C12—C17	−3.1 (2)
C2—O2—C3—C4	−174.5 (2)	C17—C12—C13—C14	0.4 (3)
O1—C3—C4—C11	7.5 (3)	C11—C12—C13—C14	179.6 (2)
O2—C3—C4—C11	−172.97 (16)	C12—C13—C14—F1	179.32 (19)
O1—C3—C4—C5	−117.2 (2)	C12—C13—C14—C15	−0.8 (3)
O2—C3—C4—C5	62.3 (2)	F1—C14—C15—C16	−179.5 (2)
C3—C4—C5—C19	−94.74 (18)	C13—C14—C15—C16	0.6 (4)
C11—C4—C5—C19	134.87 (15)	C14—C15—C16—C17	0.0 (4)
C3—C4—C5—C10	144.15 (16)	C15—C16—C17—C12	−0.3 (3)
C11—C4—C5—C10	13.77 (18)	C15—C16—C17—N2	179.6 (2)
C3—C4—C5—S1	25.0 (2)	C13—C12—C17—C16	0.1 (3)
C11—C4—C5—S1	−105.36 (15)	C11—C12—C17—C16	−179.2 (2)
C25—S1—C5—C19	−1.52 (14)	C13—C12—C17—N2	−179.83 (18)
C25—S1—C5—C4	−123.05 (14)	C11—C12—C17—N2	0.8 (2)
C25—S1—C5—C10	121.20 (13)	C18—N2—C17—C16	−177.7 (2)
C10—C6—C7—C8	54.8 (3)	C18—N2—C17—C12	2.3 (2)
C6—C7—C8—C9	−51.8 (3)	C17—N2—C18—O4	−179.8 (2)
C10—N1—C9—C8	−58.9 (2)	C17—N2—C18—C11	−4.3 (2)
C11—N1—C9—C8	177.59 (17)	N1—C11—C18—O4	58.7 (3)
C7—C8—C9—N1	52.5 (3)	C12—C11—C18—O4	179.9 (2)
C9—N1—C10—C6	64.7 (2)	C4—C11—C18—O4	−53.7 (3)
C11—N1—C10—C6	−166.38 (16)	N1—C11—C18—N2	−116.76 (18)
C9—N1—C10—C5	−167.21 (16)	C12—C11—C18—N2	4.4 (2)
C11—N1—C10—C5	−38.31 (17)	C4—C11—C18—N2	130.83 (18)
C7—C6—C10—N1	−61.1 (2)	C4—C5—C19—O3	−49.5 (3)
C7—C6—C10—C5	179.72 (18)	C10—C5—C19—O3	65.3 (2)
C19—C5—C10—N1	−104.87 (17)	S1—C5—C19—O3	−174.61 (17)
C4—C5—C10—N1	13.58 (17)	C4—C5—C19—C20	128.87 (17)
S1—C5—C10—N1	136.76 (13)	C10—C5—C19—C20	−116.35 (18)
C19—C5—C10—C6	17.4 (2)	S1—C5—C19—C20	3.7 (2)
C4—C5—C10—C6	135.84 (18)	O3—C19—C20—C21	−4.7 (4)
S1—C5—C10—C6	−100.99 (18)	C5—C19—C20—C21	177.0 (2)
C9—N1—C11—C12	−63.7 (2)	O3—C19—C20—C25	173.4 (2)
C10—N1—C11—C12	170.60 (15)	C5—C19—C20—C25	−4.8 (2)
C9—N1—C11—C4	171.82 (16)	C25—C20—C21—C22	−1.3 (3)
C10—N1—C11—C4	46.10 (17)	C19—C20—C21—C22	176.8 (2)
C9—N1—C11—C18	50.2 (2)	C20—C21—C22—C23	−0.4 (4)
C10—N1—C11—C18	−75.51 (17)	C21—C22—C23—C24	1.5 (4)
C3—C4—C11—N1	−164.31 (16)	C22—C23—C24—C25	−0.9 (4)
C5—C4—C11—N1	−35.55 (17)	C21—C20—C25—C24	1.9 (3)
C3—C4—C11—C12	73.4 (2)	C19—C20—C25—C24	−176.41 (19)
C5—C4—C11—C12	−157.80 (15)	C21—C20—C25—S1	−178.07 (17)
C3—C4—C11—C18	−44.7 (2)	C19—C20—C25—S1	3.6 (2)
C5—C4—C11—C18	84.10 (18)	C23—C24—C25—C20	−0.8 (3)
N1—C11—C12—C13	−62.1 (3)	C23—C24—C25—S1	179.14 (19)
C4—C11—C12—C13	52.5 (3)	C5—S1—C25—C20	−1.11 (17)
C18—C11—C12—C13	177.7 (2)	C5—S1—C25—C24	179.0 (2)
N1—C11—C12—C17	117.10 (18)		

### Hydrogen-bond geometry (Å, º)

**Table:**

*D*—H···*A*	*D*—H	H···*A*	*D*···*A*	*D*—H···*A*
N2—H2···O4^i^	0.87 (3)	1.96 (3)	2.834 (2)	179 (2)
C1—H1*A*···O1^ii^	0.96	2.50	3.401 (4)	156

Symmetry codes: (i) −*x*+1, −*y*+2, −*z*+1; (ii) −*x*+1, *y*−1/2, −*z*+1/2.
