# Supplementary figures and images for: Crystal structure of ethyl 5′′-fluoro-2′′,3-dioxo-6′,7′,8′,8a’-tetra­hydro-2′H,3H,5′H-di­spiro­[benzo[b]thio­phene-2,1′-indol­izine-3′,3′′-indoline]-2′-carboxyl­ate
Source: Acta Crystallogr E Crystallogr Commun. 2015 Feb 7;71(Pt 3):o156–7. doi: 10.1107/S2056989015002121 (PMC4350745; doi:10.1107/S2056989015002121)

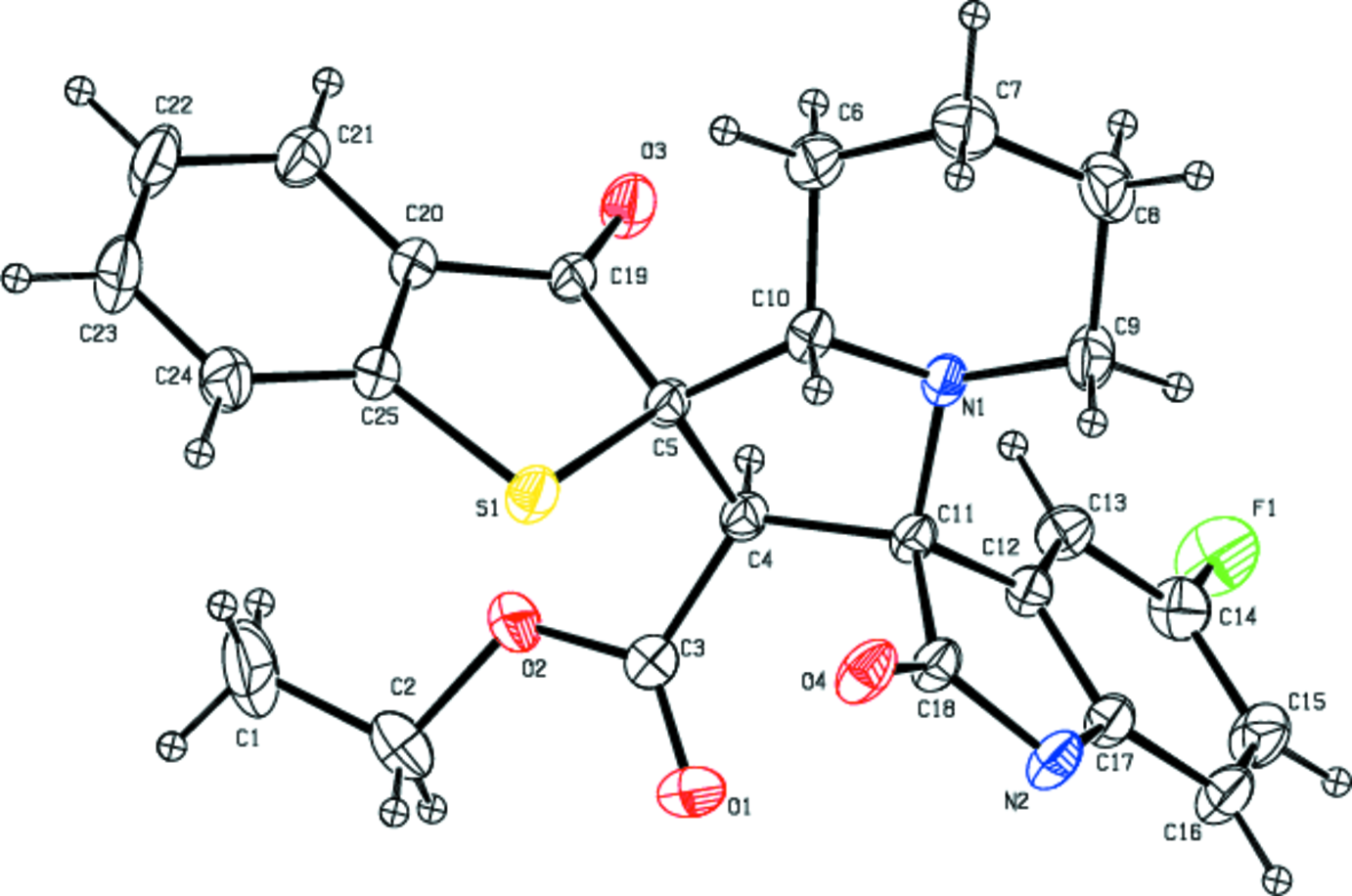

Supplement: Supplementary file 3 [file e-71-0o156-fig1.tif]

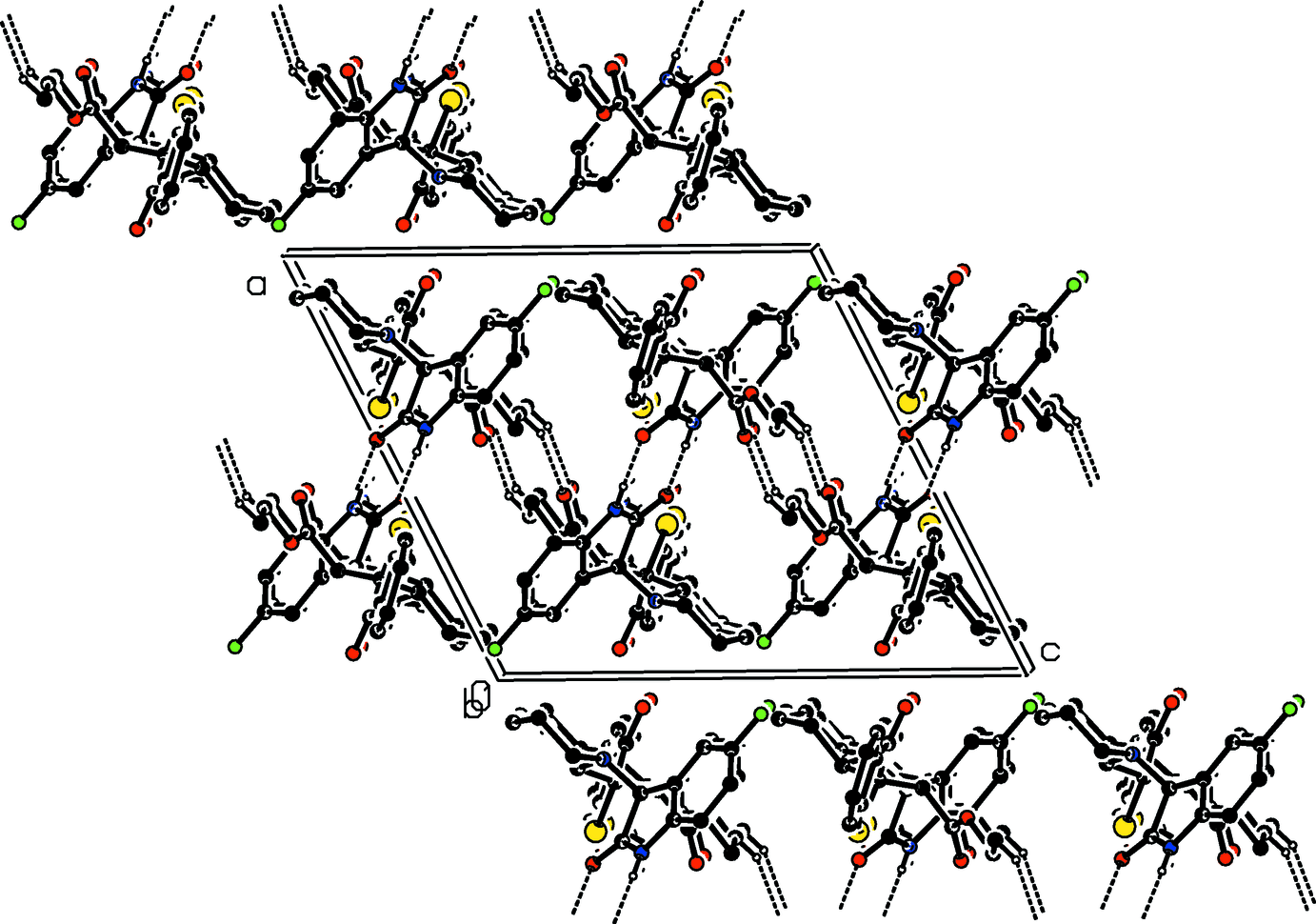

Supplement: Supplementary file 4 [file e-71-0o156-fig2.tif]
